# Impact of an Acute Care Surgery Model on the Management of Acute Appendicitis in South Korea: A Retrospective Cohort Study

**DOI:** 10.1155/2021/5522523

**Published:** 2021-03-26

**Authors:** Gun-Hee Yi, Hak-Jae Lee, Seul Lee, Jong-Hee Yoon, Suk-Kyung Hong

**Affiliations:** ^1^Department of Surgery, University of Ulsan College of Medicine, Asan Medical Center, Seoul, Republic of Korea; ^2^Division of Acute Care Surgery, Department of Surgery, University of Ulsan College of Medicine, Asan Medical Center, Seoul, Republic of Korea

## Abstract

**Background:**

The acute care surgery (ACS) system is a new model for the prompt management of diseases that require rapid treatment in patients with acute abdomen. This study compared the outcomes and characteristics of the ACS system and traditional on-call system (TROS) for acute appendicitis in South Korea.

**Methods:**

This single-center, retrospective study included all patients (aged ≥18 years) who underwent surgery for acute appendicitis in 2016 and 2018. The TROS and ACS system were used for the 2016 and 2018 groups, respectively. We retrospectively obtained data on each patient from the electrical medical records. The independent samples *t*-test and Mann–Whitney *U*-test were used for continuous and nonnormally distributed data, respectively.

**Results:**

In total, 126 patients were included. The time taken to get from the emergency room admission to the operating room, operation times, and postoperative complication rates were similar between both groups. However, the length of the hospital stay was shorter in the ACS group than in the TROS group (4.3 ± 3.2 days vs. 7.2 ± 9.6 days, *p*=0.039).

**Conclusions:**

Since the introduction of the ACS system, the length of hospital stay for surgical patients has decreased. This may be due to the application of an integrated medical procedure, such as a new clinical pathway, rather than differences in the surgical techniques.

## 1. Introduction

In America, more than 2 million patients per year are admitted for emergency surgery, and they account for 47% and 28% of mortalities and complications, respectively [1]. In patients with acute abdomen, timely surgical assessment and management are critical. In many countries, doctors overworked and patients who are in need of emergency surgery may have to wait for long periods before they can receive the appropriate treatment. However, rapid source control is important in the treatment of acute abdomen, an emergency surgical situation. If the time to operation is delayed, intraabdominal infection progresses to sepsis, and the mortality rate can be up to 36% [2, 3]. Previously, with the traditional on-call system (TROS), doctors served all patients who were admitted to the hospital, including outpatients, inpatients, and emergency room (ER) and elective surgery cases. Occasionally, surgery or treatment was delayed due to other tasks that the doctors had to perform. Therefore, the concept of the acute care surgery (ACS) system was introduced. In this model, a specialist doctor stays in the ER and quickly evaluates and treats the patients. Since acute care, trauma, and intensive care are related, the ACS system is also being developed in the trauma and surgical intensive care units (ICUs). This new model only includes patients in the ER who need timely surgical care and has been implemented at many hospitals worldwide. Acute appendicitis is the most common cause of acute abdominal pain that may require surgical treatment. According to the National Health Insurance data, the overall incidence of acute appendicitis in South Korea is 22.71 per 10000 populations per year [4]. There has been growing interest in the comparison of the outcomes of surgical and medical treatment for acute appendicitis [5]. Although medical treatment is what is currently mainly considered in the treatment of acute appendicitis, surgery is still a key in the management of acute appendicitis [6]. In this study, we compared the impact of the ACS system on the management and outcomes of general surgical emergencies, with a focus on patients who underwent appendectomy [7]. Previously, these surgeries were performed by on-call surgical fellows or residents. With the introduction of the ACS system, surgeries can be performed by more specialized surgeons. The ACS system was first implemented in August 2017 at Asan Medical Center in South Korea. They also work in shifts, performing immediate assessments and surgeries. The purpose of this single-center study was to investigate the impact of the ACS system on the outcomes of acute appendicitis.

## 2. Materials and Methods

We performed a retrospective cohort study that included all patients who underwent appendectomy at Asan Medical Center in South Korea, while the TROS (January 2016–December 2016) and ACS system (January 2018–December 2018) were being used. Patients admitted in 2017 were excluded as there was a transition period where both the ACS system and TROS were used to allow for the implementation of the ACS system. Data were retrospectively collected from the electronic medical records. The study was approved by the Institutional Review Board of Asan Medical Center (no. 2020-0783). Eligible patients were aged ≥18 years and underwent surgery for acute appendicitis. The exclusion criteria were admission to another department or admission through an outpatient clinic. The types of surgery included in the study ranged from simple appendectomy to right hemicolectomy. The primary outcome was the length of the hospital stay, which correlated with medical expenses. The secondary outcomes were the time taken to get to the operation room from the ER; ratio of daytime operations, i.e., the ratio of the number of operations that were completed between 7:00 am and 7:00 pm to the total number of completed surgeries; total operation time; conversion ratio from laparoscopy to open surgery; type of operation, which was dependent upon the severity of the acute appendicitis; and rate of postoperative complications. The time taken to get to the operation room was measured based on the time taken to enter the operating room (OR) from the time of the first visit to the ER. Postoperative complication data were obtained from the discharge summary and progression notes during admission. Postoperative complications included surgical site infection such as surgical wound infection, intraabdominal infection, ileus, and bleeding. Diagnoses were confirmed using diagnostic imaging (computed tomography). We used a grading system that was published in 2015 to evaluate the severity of the acute appendicitis based on the clinical, imaging, and laparoscopic findings (Table 1). This system was proposed by the World Journal of Emergency Surgery and uses the developments in imaging and laparoscopy techniques to reclassify acute appendicitis and allow for the optimal management of each patient [8]. Each patient's histopathology results were assessed using the hospital's pathology records to determine the histological classification of the appendix and whether there was perforation.

### 2.1. Statistical Analysis

The independent samples *t*-test and Mann–Whitney *U*-test were used for continuous and nonnormally distributed data, respectively. *p* < 0.05 was considered statistically significant. Statistical analyses were conducted using R software (version 3.3.2; R Foundation for Statistical Computing, Vienna, Austria; http://www.R-progject.org).

## 3. Results

**Figure 1:**
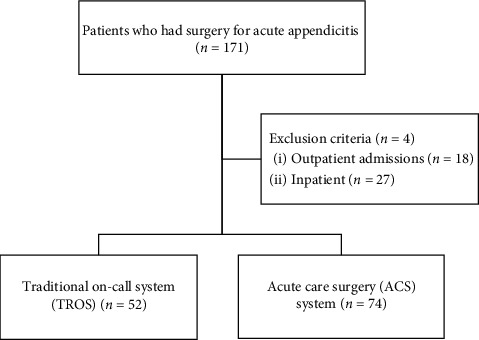
The patients who underwent surgery for acute appendicitis over the two years (2016 and 2018).

During the 2-year study period, 171 patients underwent appendectomy, 45 of whom were outpatients or in-hospitalized patients. The TROS and ACS system were used for 52 and 74 patients, respectively (Figure 1).

There were no significant differences in patient demographics between the 2 groups, and the Charlson comorbidity index showed no statistically significant differences in the underlying diseases between the 2 groups (TROS: 1.4 ± 2.0 vs. ACS: 1.3 ± 18, *p*=0.769). There were no statistically significant differences in the grades of acute appendicitis (*p*=0.095), but the grade tended to be higher in the post-ACS group than in the post-TROS group. When appendicitis grades 3 and 4 were analyzed separately, they were significantly higher in the post-ACS group than in the post-TROS group (TROS: 32.7% vs. ACS: 54.1%, *p*=0.029, Table 2).

There were significant differences between the groups regarding the time taken to get from the ER to the OR, ratio of daytime operations, total operation time, and laparoscopic operation and postoperative complication rates. More surgeries were performed during the day in the post-ACS group than in the post-TROS group, but the results were not statistically significant (TROS: 46.2% vs. ACS: 63.5%, *p*=0.080). The surgeries were divided into 4 types (simple appendectomy, partial cecectomy, ileocecal resection, and right hemicolectomy). Compared to the post-TROS group, a greater number of complicated operations than simple appendectomies were performed and the length of the hospital stay was shorter in the post-ACS group (TROS: 11.5% vs. ACS: 31.1% and TROS: 7.2 ± 9.6 days vs. 4.3 ± 3.2 days; *p*=0.037 and *p*=0.039, respectively; Table 3).

## 4. Discussion

Acute appendicitis is the most common surgical emergency seen in emergency departments. Timely access to acute emergency surgery is important for patients' outcomes, and there are numerous factors that contribute to poor outcomes such as a shortage of surgeons, inadequate numbers of emergency ORs, and the lack of an emergency team [9]. The ACS system was introduced to serve as a solution for the problem of a lack of a dedicated emergency surgeon. The United States introduced it in the early 2000s, and it is systematically being implemented in several areas, including trauma centers and surgical ICUs. Recently, several studies on the impact of the implementation of the ACS system have been published; one study reported that, compared to the TROS, the ACS system lowered the mortality and complication rates and reduced the time to operation and financial costs. A meta-analysis of 27 studies that included 744000 patients with emergency surgical diseases such as acute appendicitis, acute cholecystitis, and inguinal hernia reported that the application of the ACS system had improved the clinical and financial outcomes for emergency general surgery [10]. Additionally, according to several previous studies, when comparing and analyzing general surgical conditions such as acute appendicitis and acute cholecystitis, the times taken to get from the ER to the OR and postoperative complication rates were reduced after the introduction of the ACS system [11–13]. In our study, with the implementation of the ACS, the median length of hospital stay was reduced by 3 days; however, this was not explained by the introduction of the ACS system alone. Before the introduction of the ACS system, the clinical pathway (CP) that was used to treat hospitalized patients was not uniform and patients were managed by different surgeons from various surgical divisions; however, with the introduction of this system, we created a unified CP for the management of acute appendicitis. To prevent unnecessary antibiotic abuse, delay of diet proceedings, and unnecessary X-ray and laboratory evaluations, the admission and discharge periods and the use of antibiotics according to the grade of the acute appendicitis were determined in advance. This was a difficult task to perform with the TROS.

The times taken to get from the ER to the OR, which were expected to have an effect in this study, did not differ between the 2 groups. After the introduction of the ACS system, the ACS surgeon is always on-call, which allows for surgical decisions to be made, as well as for the treatment plan to be determined as soon as possible. However, in order to perform the surgeries, various components relating to personnel and materials such as the OR, anesthesiologist, and nurse, in addition to the surgeon, must be prepared on-call. In order to shorten the time taken to get from the ER to the OR, it is necessary to prepare the on-call team and facilities (such as the anesthesiologist, OR, and scrub nurse), as well as the ACS surgeon, for emergency surgery.

Although, at our center, complicated operations, such as right hemicolectomy, were performed to a greater extent in the post-ACS group, there were no differences in the rates of the postoperative complications between the 2 groups. Regarding acute appendicitis, only simple appendectomies were performed in more than two-thirds of the cases, the possibilities of severe complications were low, and the course of the disease was benign, so acute appendicitis itself is not considered a disease that greatly affects the mortality or morbidity of the patients. We considered that this may have a greater effect on outcomes such as the length of hospital stay than the rates of postoperative complications or mortality, as in our study.

This study has some limitations. First, it was a retrospective study, which may have been a source of selection bias. Second, the medical environment in South Korea is unique. In South Korea, since medical care is relatively inexpensive, patients tend to remain hospitalized for no specific reason, which limits the ability to base the evaluation of the length of hospital stay solely on the effect of the ACS system. Third, our study did not analyze the hospital costs. Other studies reported that the ACS system resulted in a reduction in the hospital costs [10, 12]. However, in South Korea, medical expenses for acute appendicitis are calculated and paid for in advance, so there are no significant differences in the medical expenses. Therefore, we did not perform a cost analysis in this study.

## 5. Conclusion

This study is significant as it is one of the early studies that explores the effects of the implementation of the ACS system in South Korea. The length of hospital stay was significantly reduced, which may be attributed to the introduction of the ACS system and other factors, such as the changes to the CP. In future, to confirm the effectiveness of the ACS system, more research should be conducted on diseases that require emergency general surgery other than acute appendicitis.

## Figures and Tables

**Table 1:** New grading system for acute appendicitis (2015), World Journal of Emergency Surgery (WJES).

Noncomplicated acute appendicitis	
Grade 0, appendix appears normal (endoappendicitis/periappendicitis)	
Grade 1, inflamed appendix (hyperemia, edema ± fibrin without or with little pericolic fluid)	

Complicated acute appendicitis	
Grade 2, necrosis	A: segmental necrosis (without or with little pericolic fluid)
	B: base necrosis (without or with little pericolic fluid)
Grade 3, inflammatory tumor	A: phlegmon
	B: abscess measuring <5 cm without peritoneal free air
Grade 4, perforated	Diffuse peritonitis with or without peritoneal free air

**Table 2:** Demographic characteristics of the patients with appendicitis (n = 126).

	TROS group (*n* = 52)	Post-ACS group (*n* = 74)	*p* value
Age (years)	51.8 ± 19.8	54.1 ± 19.5	0.501

Male (*n*, %)	31 (59.6%)	41 (55.4%)	0.774

Underlying disease (*n*, %)			
DM	10 (19.2%)	12 (16.2%)	0.841
HTN	12 (23.1%)	25 (33.8%)	0.271
CVA	5 (9.6%)	7 (9.5%)	1.000
CAD	5 (9.6%)	7 (9.5%)	1.000
CKD	5 (9.6%)	3 (4.1%)	0.374
Malignancy	10 (19.2%)	17 (23.0%)	0.777

Charlson comorbidity index	1.4 ± 2.0	1.3 ± 1.8	0.769

Appendicitis grade (*n*, %)			0.095
1	29 (55.8%)	30 (40.5%)	
2	6 (11.5%)	4 (5.4%)	
3	3 (5.8%)	10 (13.5%)	
4	14 (26.9%)	30 (40.5%)	

Perforated appendicitis (grades 3 and 4; *n*, %)	17 (32.7%)	40 (54.1%)	0.029^*∗*^

Values are expressed as mean ± standard deviation or number and percentage (*n*, %). TROS, traditional on-call system; ACS, acute care surgery; DM, diabetes mellitus; HTN, hypertension; CVA, cerebrovascular accident; CAD, coronary artery disease; CKD, chronic kidney disease. ^*∗*^*p* values of <0.05 were considered statistically significant.

**Table 3:** Comparison of clinical outcomes according to the system used for patient management (*n* = 126).

	TROS group (*n* = 52)	Post-ACS group (*n* = 74)	*p* value

Time taken to get from the ER to the OR (min)	1060.1 ± 1341.0	972.1 ± 1044.2	0.693
Daytime operations (*n*, %)	24 (46.2%)	47 (63.5%)	0.080
Total operation time (min)	87.4 ± 39.7	99.6 ± 59.1	0.168
Laparoscopic operation (*n*, %)	43 (82.7%)	68 (91.9%)	0.197
Open conversion rate (*n*, %)	4 (9.3%)	1 (1.5%)	0.142
Operation type (*n*, %)			0.037^*∗*^
Simple appendectomy	46 (88.5%)	51 (68.9%)	
Partial cecectomy	2 (3.8%)	12 (16.2%)	
Ileocecal resection	3 (5.8%)	4 (5.4%)	
Right hemicolectomy	1 (1.9%)	7 (9.5%)	
Length of hospital stay (days)	7.2 ± 9.6	4.3 ± 3.2	0.039^*∗*^
ICU admission (*n*, %)	4 (7.7%)	2 (2.7%)	0.384
Postoperative complications (*n*, %)	7 (13.5%)	9 (12.2%)	1.000

Values are expressed as mean ± standard deviation or number and percentage (*n*, %). TROS, traditional on-call system; ACS, acute care surgery; ER, emergency room; OR, operating room; ICU, intensive care unit. ^*∗*^*p* values of <0.05 were considered statistically significant.

## Data Availability

The datasets used and/or analyzed during the current study are available from the corresponding author upon request.

## Abbreviations

ACS: Acute care surgery
TROS: Traditional on-call system
ER: Emergency room
OR: Operating room
CP: Clinical pathway
ICU: Intensive care unit
DM: Diabetes mellitus
HTN: Hypertension
CVA: Cerebrovascular accident
CAD: Coronary artery disease
CKD: Chronic kidney disease.
